# Neutrophils as Orchestrators in Tumor Development and Metastasis Formation

**DOI:** 10.3389/fonc.2020.581457

**Published:** 2020-12-10

**Authors:** Lydia Kalafati, Ioannis Mitroulis, Panayotis Verginis, Triantafyllos Chavakis, Ioannis Kourtzelis

**Affiliations:** ^1^ Institute for Clinical Chemistry and Laboratory Medicine, University Hospital and Faculty of Medicine, Technische Universität Dresden, Dresden, Germany; ^2^ National Center for Tumor Diseases, Partner Site Dresden and German Cancer Research Center, Heidelberg, Germany; ^3^ Department of Hematology and Laboratory of Molecular Hematology, Department of Medicine, Democritus University of Thrace, Alexandroupolis, Greece; ^4^ University of Crete, School of Medicine, Heraklion, Greece; ^5^ Institute of Molecular Biology and Biotechnology, Foundation for Research and Technology-Hellas, Heraklion, Crete, Greece; ^6^ York Biomedical Research Institute, Hull York Medical School, University of York, York, United Kingdom

**Keywords:** neutrophil, tumor, metastasis, immune modulation, cancer-dependent granulopoiesis, trained immunity, innate immune memory, trained granulopoiesis

## Abstract

Several lines of clinical and experimental evidence suggest that immune cell plasticity is a central player in tumorigenesis, tumor progression, and metastasis formation. Neutrophils are able to promote or inhibit tumor growth. Through their interaction with tumor cells or their crosstalk with other immune cell subsets in the tumor microenvironment, they modulate tumor cell survival. Here, we summarize current knowledge with regards to the mechanisms that underlie neutrophil–mediated effects on tumor establishment and metastasis development. We also discuss the tumor-mediated effects on granulopoiesis and neutrophil precursors in the bone marrow and the involvement of neutrophils in anti-tumor therapeutic modalities.

## Introduction

Neutrophils comprise the majority of leukocytes in humans and are considered the first immune cell population to respond against infectious and inflammatory insults (1–5). This innate immune cell type fine-tunes the armament of host defense through modulating phagocytosis and intracellular killing of pathogens, release of proteases and antimicrobial peptides from their granules, as well as formation of neutrophil extracellular traps (NETs) (6–9). In addition, neutrophils mediate interactions between innate and adaptive immunity by shaping antigen presentation (10, 11) and the production of chemokines and cytokines (12, 13). The generation of neutrophils from their myeloid precursors, designated as granulopoiesis, takes place in the bone marrow, where neutrophils accumulate until they are released in the circulation in a timely and tightly controlled process (14–16). Billions of neutrophils are produced daily under steady state conditions (17). However, certain types of stress such as exposure to inflammatory or infectious agents or cancer result in emergency granulopoiesis that induces a rapid increase in neutrophil production (1, 18). Neutrophils may have gained less attention than other immune cells in the study of anti-tumor immunity due to their relatively short lifespan. However, neutrophil survival is much longer than initially thought; they can remain alive for at least 5 days in the circulation (19). In addition, neutrophils are generated in high numbers daily and recent findings point to substantial neutrophil heterogeneity (20). Recent evidence thus suggests their involvement in shaping of pro-tumor and anti-tumor responses (21). For instance, neutrophils promote the formation of the pre-metastatic niche and neutrophils from mice with early-stage tumors display increased migratory activity compared to neutrophils from tumor-free animals (22). On the other hand, neutrophils with certain phenotypic characteristics have been associated with enhanced tumor suppression. Specifically, a subset of tumor-associated neutrophils from patients diagnosed with early-stage human lung cancer bears antigen-presentation activity thereby facilitating anti-tumor immunity (23). To further support the dual and context-dependent role of neutrophils in tumors, low-density neutrophils have been shown to be more immunosuppressive and to promote cancer progression as compared to high-density neutrophils (24). In addition, neutrophil plasticity and localization at the tumor site depends not only on intrinsic cues, but also on the type and the stage of the tumor (25). Here, we discuss the neutrophil-dependent mechanisms that may affect suppression or progression of primary tumors and establishment of metastasis.

## Neutrophils Contribute to Tumor Progression

**Figure 1:**
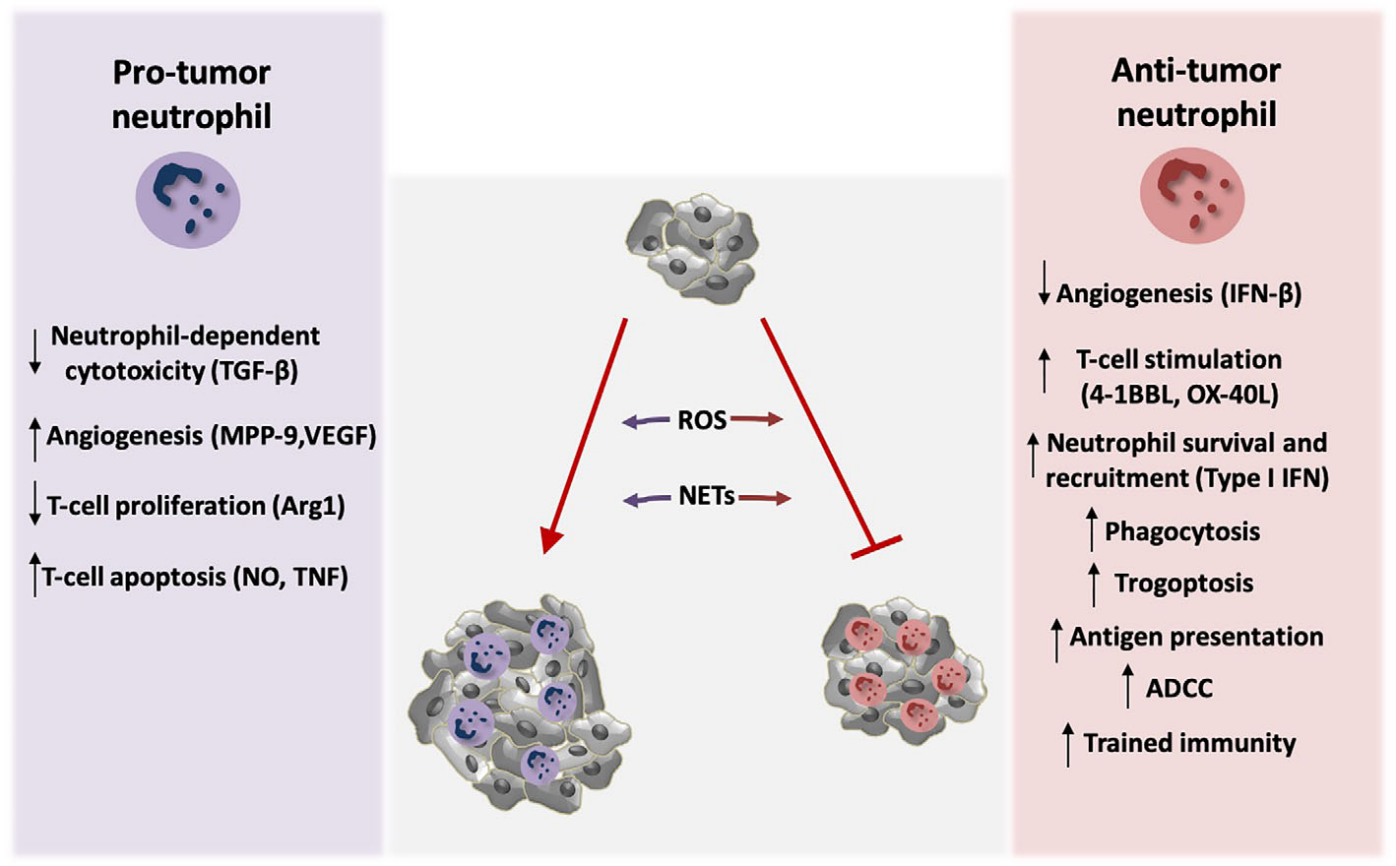
Neutrophil activity modulates tumor growth. Neutrophils exert both tumor-promoting and tumor suppressive functions. TGF-β signaling induces polarization of neutrophils towards pro-tumor phenotype, by blocking direct neutrophil-dependent tumor cell killing. Activation of tumor angiogenesis is stimulated by the production of the neutrophil-derived pro-angiogenic factors MMP-9 and VEGF, whereas endogenous IFN-β downregulates these factors resulting in inhibition of angiogenesis. Neutrophils also modulate anti-tumor T-cell responses. Arginase 1 (Arg1) secretion by neutrophils inhibits T-cell proliferation. Moreover, nitric oxide (NO), and tumor necrosis factor (TNF) derived by neutrophils induce T-cell apoptosis. On the contrary, neutrophils activate T-cell proliferation and anti-tumor function through the production of co-stimulatory molecules such as 4-1BBL and OX-40L. Type I IFN signaling induces neutrophil-mediated tumor suppression by increasing their survival and recruitment in the tumor. Neutrophils can also kill tumor cells directly *via* antibody dependent cell-mediated cytotoxicity (ADCC) and trogoptosis. In addition, they phagocytose tumor cells and mediate antigen presentation resulting in enhanced anti-tumor immunity. Induction of trained immunity has also been described to promote neutrophil-dependent tumor suppression. ROS production and formation of NETs by neutrophils play a dual role in tumor expansion in a context-dependent manner. Specifically, neutrophils produce ROS that leads to genetic instability and carcinogenesis. On the other hand, ROS can mediate tumor cell killing. NETs contain MMP-9, cathepsin G, and neutrophil elastase that promote tumor growth, but in parallel NET formation primes T-cells and leads to enhanced anti-tumor responses.

Emerging evidence suggests that neutrophils modulate cancer-associated inflammation. Importantly, inflammation is a hallmark of cancer (26) and represents an essential contributor to the development of many tumors (27). Neutrophils are present in several types of human tumors and neutrophil accumulation in certain tumors is correlated with poor prognosis (27–29). Inflammatory mediators can affect plasticity of tumor-associated neutrophils and polarize them towards either pro-tumor or anti-tumor phenotype (25, 30, 31) (**Figure 1**). Fridlender et al. have shown that blockade of transforming growth factor β (TGFβ) signaling leads to increased neutrophil influx in the tumor. More importantly, these infiltrated neutrophils acquire an anti-tumor phenotype suggesting that TGFβ polarizes neutrophils toward a pro-tumor phenotype (30). In addition, the pro-tumoral role of neutrophils has been associated with promotion of angiogenesis (32, 33). Tumor expansion requires the development of new blood vessels that ensure sufficient supply of oxygen and nutrients. Tumor–infiltrating neutrophils are a source of matrix metalloprotease 9 (MMP-9) promoting remodeling of extracellular matrix (ECM) and neovascularization (34). Along this line, reduction of tumor-associated angiogenesis was observed after neutrophil depletion (35). Neutrophils also produce the major pro-angiogenic factor vascular endothelial growth factor (VEGF) regulating tumor-associated angiogenesis (36).

The immunosuppressive function of neutrophils may also contribute to their tumor-promoting function. Neutrophils mediate the suppression of CD8^+^ T cell proliferation and activation by inducing their apoptosis in a manner dependent on nitric oxide and TNF production (37). Furthermore, upregulation of arginase 1 in neutrophils inhibits T-cell proliferation (36, 38) thereby promoting immunosuppression and tumor evasion. Consistently, neutrophil depletion in a mouse model of lung cancer resulted in increased CD8^+^ T cell activation and in decreased tumor burden (30). Furthermore, neutrophils exert their protumorigenic activity by releasing oxygen and nitrogen free radicals that promote genetic instability and carcinogenesis (39–41).

Neutrophils are able to generate neutrophil extracellular traps (NETs). These structures contain extracellular fibers composed of chromatin, histones, and other proteins (42, 43). Except from their established role in host-pathogen interactions (42), NETs modulate cancer-associated procoagulant activity (44) and promote tumor growth (45, 46) by including tumor-promoting components such as MMP-9, cathepsin G (47) and neutrophil elastase (45, 46). In addition, presence of NETs in patients diagnosed with cancer has been associated with poor prognosis (48) and blockade of IL17-mediated NET generation resulted in increased responsiveness to immune checkpoint blockade in pancreatic ductal adenocarcinoma (49).

## Tumor-Suppressive Activity of Neutrophils

Besides their pro-tumorigenic role, neutrophils can function as tumor suppressors boosting anti-tumor activity (**Figure 1**). Neutrophils have the capacity to generate reactive oxygen species (ROS) by the NADPH oxidase complex and mediate anti-tumor responses (50–52). In an autochthonous mouse tumor model, tumor oxygenation levels differentially affected neutrophil function, and inhibition of tumor hypoxia was associated with enhanced neutrophil dependent-tumor cell killing as a result of ROS production (53).

Antibody dependent cell-mediated cytotoxicity (ADCC) represents another way by which neutrophils may kill tumor cells. In particular, neutrophils express several Fc receptors (FcRs), such as FcγRI (CD64), FcγRIIa (CD32), FcγRIIIa (CD16a), and FcγRIIIb (CD16b) that recognize tumor cell-specific antibodies and mediate ADCC (52, 54, 55). In addition, neutrophil phagocytosis of opsonized tumor cells enhances anti-tumor activity (56) as shown with human tumor cells (57). Neutrophil trogoptosis has been described to exert tumor suppressive activity (58). Specifically, neutrophils target and destroy tumor cells that are opsonized with therapeutic monoclonal antibodies in a process that involves tumor cell lysis mediated by trogocytosis (58–60).

Type I interferons contribute to the anti-tumor effects of neutrophils. Endogenous interferon-β (IFN-β) has been shown to inhibit angiogenesis by downregulating the proangiogenic factors VEGF and MMP-9 in tumor-infiltrating neutrophils (61). Consistently, type I IFN signaling mediates neutrophil-dependent anti-tumor activity by modulating neutrophil survival and recruitment into the tumor (62, 63). Furthermore, neutrophils contribute to the activation of the IFN-γ pathway that enhances anti-tumor activity mediated by the activity of CD4^-^CD8^-^ unconventional αβ T-cells. In agreement with these findings, neutrophil infiltration in certain types of tumors was linked to better clinical outcome (64). Interestingly, NET formation has been also associated with inhibition in tumor growth. Specifically, NETs prime T-cells and play potential role in cancer immunoediting and enhancement of antitumor responses (48, 52).

Up-regulation of antigen presentation can mediate neutrophil–dependent anti-tumor activity. Beauvillain et al. have shown that neutrophils process and present antigens to T-cells (65), thereby enhancing T-cell mediated antitumor responses (66, 67). Along the same line, a subset of neutrophils from patients diagnosed with early-stage human lung cancer has exhibited up-regulated antigen-presenting activity. This neutrophil subpopulation originates from specific bone marrow progenitors upon exposure to IFNγ and GM-CSF signaling (23). Neutrophils can additionally promote T-cell responses *via* the production of the co-stimulatory molecules 4-1BBL and OX-40L, which enhance proliferation and activation of CD4^+^ and CD8^+^ T-cells and increase their cytotoxic capacity in a model of lung cancer (68).

## Neutrophils Modulate Metastatic Dissemination of Cancer Cells

**Figure 2:**
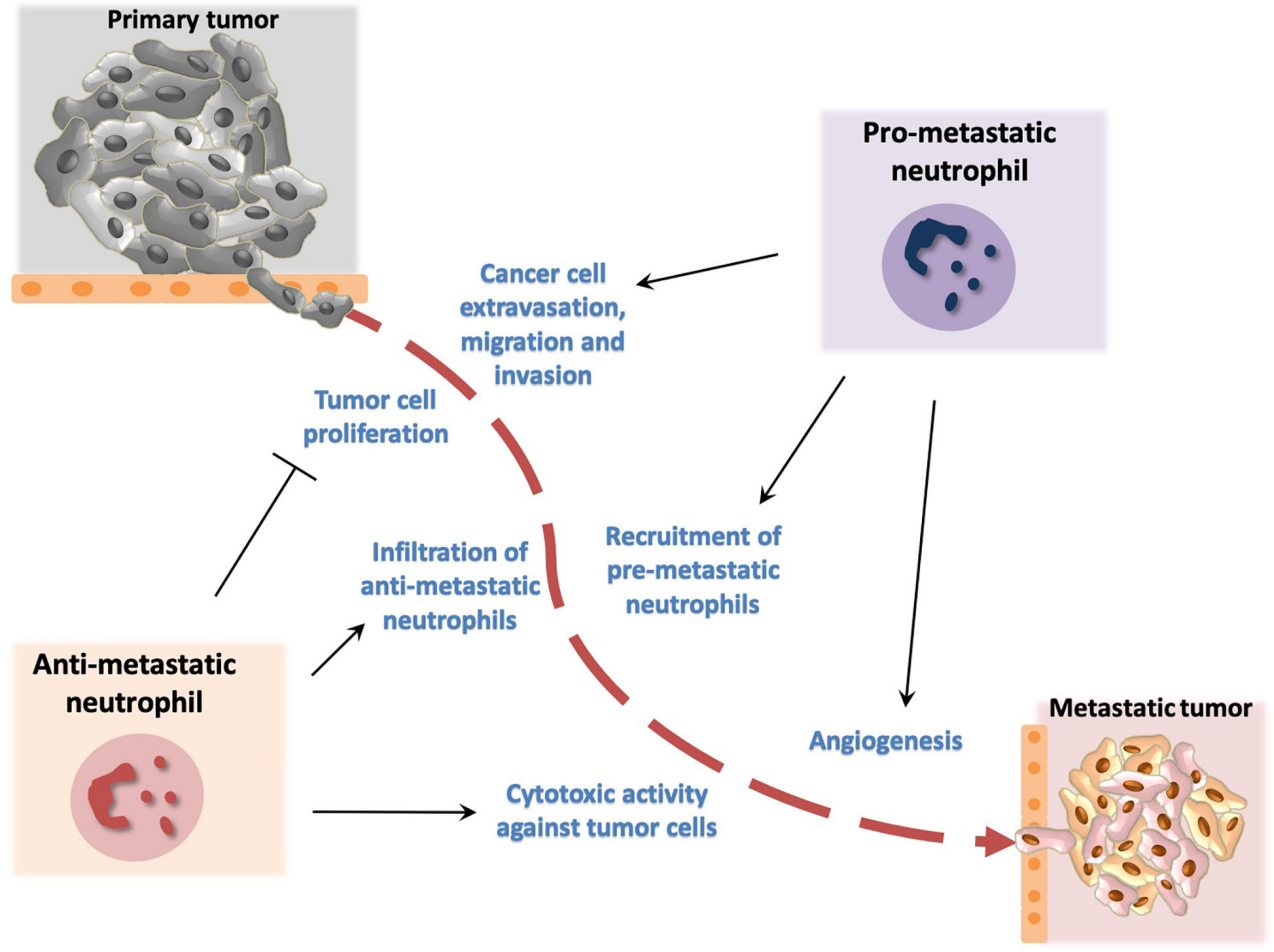
Dual role of neutrophils in metastasis development. Neutrophils promote metastasis by facilitating cancer cell extravasation from primary tumor, migration to the metastatic site and invasion in secondary tumors. In addition, neutrophils promote angiogenesis that has been associated with development of metastasis. Neutrophil activity has been also implicated in inhibition of metastasis. In particular, neutrophils have been described to block cancer cell proliferation and to exert cytotoxic activity thereby affecting tumor cell survival.

Detachment and escape of tumor cells from the primary tumor represents the initial step of metastasis that is followed by intravasation into the blood and lymphatic system, extravasation, and colonization of tumor cells to distant organs or draining lymph nodes (69). Metastasis is associated with increased mortality (70, 71). Neutrophils affect not only the growth of primary tumors but also orchestrate the metastatic potential of cancer cells (72, 73). Specifically, large body of evidence supports that neutrophils contribute to the initiation phase of metastatic dissemination (69, 74) (**Figure 2** and **Supplementary Table 1**). In addition, primary tumor growth has been associated with accumulation of neutrophils in distant organs before the arrival of the disseminated tumor cells to the site designated as premetastatic niche (75–77). Along the same line, primary tumor cells release factors that render distinct sites more prone to become metastatic sites. The accumulation of neutrophils at these sites is dependent on the growth factor granulocyte-colony stimulating factor (G-CSF) in several tumor models (75, 76, 78). G-CSF promotes the pro-metastatic phenotype of neutrophils by inducing BV8 expression in neutrophils (75, 79) that in turn enhances angiogenesis and cancer cell migration (80, 81). Additionally, neutrophils cooperate with γδ T-cells, in an interleukin-17/G-CSF dependent manner to facilitate breast cancer metastasis. Depletion of neutrophils in an experimental model of metastatic breast cancer in mice led to a decrease of metastatic burden in both lymph nodes and lungs (75). The interaction of neutrophils with endothelial cells also enhances metastasis by facilitating tumor cell extravasation into the circulation (82–86).

Oncostatin M functions as a pro-angiogenic factor that promotes metastasis. In particular, co-culture experiments have demonstrated that exposure of neutrophils to granulocyte-macrophage colony stimulating factor (GM-CSF) results in production of oncostatin M that contributes to metastasis in a model of breast cancer (87). The glycosaminoglycan hyaluronan, a major component of the ECM that is produced by various types of tumor cells, activates neutrophils through TLR4 signaling and promotes malignant cell migration (88). Furthermore, macrophage migration inhibitory factor (MIF) promotes neutrophil chemotaxis that in turn leads to increased migratory capacity of tumor cells in an *in vitro* model of head and neck cancer (89). Another *in vitro* study using a model of renal cell carcinoma revealed higher recruitment of neutrophils towards tumor cells that was associated with enhanced cancer cell migration and invasion in a manner dependent on a VEGF/hypoxia inducible factor 2a signaling (90). A metastasis-promoting role for neutrophils has been observed in a model of bladder cancer, in which infiltrating neutrophils contribute to cancer cell invasion *via* mediating an upregulation of androgen receptor signals (91).

Besides their impact on primary tumor growth, NETs may contribute to metastasis and blockade of NET formation resulted in decreased tumor metastasis in mice (92, 93).

In agreement with the plasticity and context-specific phenotype of neutrophils, some neutrophil depletion studies have resulted in increased incidence of metastasis (94). Specifically, the chemokine CCL2 has been shown to promote activation of neutrophils towards an anti-metastatic phenotype in a mouse model of lung metastasis. These neutrophils acquired tumor cytotoxic activity that was mediated by production of ROS and H_2_O_2_ (94). Along the same line, the proto-oncogene MET has been linked to neutrophil-dependent inhibition of primary tumor growth and metastasis formation. Deletion of *Met* in neutrophils resulted in decreased neutrophil infiltration and nitric oxide–dependent tumor cell killing and reduced metastasis (95). In addition, thrombospondin 1 (Tsp1) that is derived by Gr1^+^ bone marrow myeloid cells may restrain metastasis. Consistently, deficiency in proteases that mediate Tsp1 degradation was associated with decreased metastasis dissemination (96).

## The Tumor-Related Impact on Neutrophil Production in The Bone Marrow

Aberrant myelopoiesis is a hallmark of cancer (97). Tumor-associated inflammation reprograms hematopoiesis in the bone marrow *via* acting on hematopoietic stem and progenitor cells (HSPCs) (98). These cells are responsible for the maintenance of hematopoiesis and give rise to all hematopoietic cells through several steps of differentiation (14). Inflammatory stimuli, including those associated with cancer, activate HSPCs, thus promoting their proliferation and myeloid cell priming (16, 99). Studies in patients with cancer (100) and tumor-bearing mice (101) have demonstrated that the tumor environment drives a myeloid bias in HSPCs resulting in enhanced production of cells of the myeloid lineage, at the expense of cells of lymphoid lineage, which has been correlated with disease prognosis in different types of malignancy (102, 103). Circulating hematopoietic progenitor subsets were increased in patients with cancer compared to age-matched healthy subjects (100). Interestingly, the same study has reported enhanced frequency of granulocyte-macrophage myeloid progenitors (GMPs) in the circulation of patients with cancer, further suggesting the myeloid priming of hematopoiesis (100). Increased frequency of myeloid-biased HSPCs residing in the spleen has also been reported in tumor-bearing mice (101). These cells were responsive to the myelopoiesis-driving growth factor GM-CSF, which resulted in production of myeloid cells with pro-tumorigenic properties (101).

Further studies have implicated the myeloid lineage growth factors GM-CSF and G-CSF in the generation of increased numbers of neutrophils in cancer. In a mouse model of invasive breast carcinoma, tumor cell–derived G-CSF can activate bone marrow hematopoietic progenitors, driving myeloid differentiation, and production of neutrophils with T-cell suppressive properties (78). G-CSF can induce mobilization of granulocytes from the bone marrow, which in turn results in their accumulation to distal tissues, supporting metastasis (76). A study in a mouse model of pancreatic ductal adenocarcinoma has shown that tumor-derived GM-CSF regulates the generation of immunosuppressive Gr1^+^CD11b^+^ myeloid cells (104). Mutations in the oncogenic gene KRAS were shown to drive the increased production of GM-CSF by pancreatic ductal endothelial cells, thus further fueling myelopoiesis (105). Except from the myeloid lineage growth factors, TNF supports tumor-associated aberrant myelopoiesis. TNF released by activated CD4^+^ T cells in tumor-bearing mice drives emergency myelopoiesis and generation of both monocytic and granulocytic myeloid cells with immunosuppressive properties (106).

Recent studies have identified unipotent neutrophil precursors that expand in the bone marrow and circulation of tumor-bearing mice (107, 108). These neutrophil precursors have immunosuppressive and tumor-promoting characteristics, as shown in a mouse melanoma model (107). Such circulating neutrophil precursors were also identified in patients with melanoma (107). Using a xenograft osteosarcoma model, it was demonstrated that these neutrophil precursors promote tumor growth (107). Taken together, cancer is associated with aberrant myelopoiesis, which usually results in the generation of neutrophil precursors and neutrophils with tumor-promoting potential.

## Neutrophil-Associated Anti-Tumor Therapeutic Approaches

Given their involvement in the shaping of pro-tumor or anti-tumor activity, neutrophils may serve as a therapeutic target in the context of tumor progression. For instance, blockade of the recruitment of pro-tumorigenic neutrophils into tumor may represent a promising strategy against tumor expansion (109). Along the same line, administration of a neutralizing antibody against the neutrophil chemokine interleukin 8 (IL-8) that can be secreted by tumor cells resulted in decreased primary tumor growth and metastasis in models of melanoma and lung cancer (110). Inhibition of the CXC chemokine receptor 2 (CXCR2), a major receptor for IL-8, also led to decreased neutrophil presence in tumors and was associated with tumor suppression (111). Additionally, blockade of CXCR2 demonstrated anti-metastatic effect and led to increased efficacy of either immunotherapy in a model of pancreatic ductal adenocarcinoma (112) or chemotherapy in breast carcinoma (113). Neutrophil depletion led to increased sensitivity to radiation therapy in a mouse model of sarcoma (114). The ratio of CD8^+^ T-cells to neutrophils within the tumor of patients with non–small cell lung cancer has been suggested as a marker indicative of immune checkpoint inhibitor efficacy (115). In addition, accumulation of Gr1^+^CD11b^+^ cells that is mediated by G-CSF–induced mobilization (116, 117) or not (118) was associated with decreased tumor responsiveness after therapeutic inhibition of angiogenesis.

On the other hand, there are reports suggesting a beneficial impact of neutrophils by promoting tumor elimination. Neutrophils were shown to mediate T-cell anti-tumor activity in early stages of human lung cancer (68). Additionally, neutrophils derived from healthy donors have demonstrated tumor cell killing potential (119). Combination of radiation therapy with G-CSF administration has also resulted in neutrophil-dependent anti-tumor immunity as shown in syngeneic mouse tumor models (120).

Manipulation of the phenotype of tumor-associated neutrophils can be exploited as a potential anti-tumor therapeutic approach. Inhibition of TGFβ signaling promotes reprograming of tumor-associated neutrophils, shifting their actions from pro-tumor to anti-tumor (30). Deficiency of TGFβ signaling in myeloid cells has also resulted in inhibition of metastasis that was associated with enhanced anti-tumor immunity (121). Additionally, priming of tumor-associated neutrophils with IFNγ and TNF contributed to alterations in the polarization of neutrophils rendering them from tumor promoters to tumor suppressors (122).

Moreover, recent evidence suggests that trained immunity may confer anti-tumor properties in neutrophils. Trained immunity represents memory of the innate immune system (123). In particular, exposure of innate immune cells to certain stimuli, such as the microbial component β-glucan or the Bacillus Calmette–Guérin vaccine leads to enhanced responsiveness to subsequent homologous or heterologous triggers (123, 124). Epigenetic reprograming of myeloid cells and their progenitors in the bone marrow represent major components of innate immune memory (125–127). Trained innate immunity may boost neutrophil-dependent tumor suppression. Specifically, β-glucan-induced trained immunity led to epigenetic reprograming of granulopoiesis towards generation of neutrophils with an anti-tumor phenotype. Trained granulopoiesis was mediated by type I IFN signaling, while the tumor suppressive activity of “trained” neutrophils was mediated by enhanced ROS production. The therapeutic potential of ‘trained’ neutrophils was confirmed by the decreased tumor growth in mice that received neutrophils from β-glucan–treated donor mice (128).

## Concluding Remarks

Myeloid cells play an important role in the modulation of tumor growth. Neutrophils not only respond against pathogens and inflammatory stimuli, but also orchestrate tumor-associated immune responses. Different polarization signals can affect neutrophil plasticity and in turn lead to either promotion or suppression of primary tumors or metastasis. Neutrophils can affect cancer progression by interacting directly with tumor cells or indirectly with other immune cell types. Importantly, tumor-associated inflammation has a substantial impact in neutrophil production in the bone marrow that is a key determinant in tumor growth.

Given the increasing need to optimize the efficacy of tumor immunotherapy, a better understanding of the granulopoiesis- and neutrophil-related mechanisms that shape anti-tumor immunity is required.

## Author Contributions

LK, IM, PV, TC, and IK have made substantial, direct, and intellectual contribution to the manuscript. All authors contributed to the article and approved the submitted version.

## Funding

IK is supported by a start-up funding from the Hull York Medical School of University of York and the Royal Society research grant RGS\R2\202032.

## Conflict of Interest

The authors declare that the research was conducted in the absence of any commercial or financial relationships that could be construed as a potential conflict of interest.

## Supplementary Material

Supplementary Table 1 The impact of neutrophil activity on metastasis formation.
